# Cytology *vs* molecular analysis for the detection of head and neck squamous cell carcinoma in oesopharyngeal brush samples: a prospective study in 56 patients

**DOI:** 10.1038/sj.bjc.6600953

**Published:** 2003-05-27

**Authors:** S Temam, M Trassard, G Leroux, J Bosq, B Luboinski, G Lenoir, J Bénard, F Janot

**Affiliations:** 1 Head and Neck Surgery Department, Institut Gustave-Roussy, 39 rue Camille Desmoulins, Villejuif 94805, France; 2 Pathology Department, Centre René Huguenin, St Cloud 92210, France; 3 Pathology Department, Institut Gustave-Roussy, Villejuif 94805, France; 4 Genetics Department, Institut Gustave-Roussy, Villejuif 94805, France

**Keywords:** genetic markers, microsatellite repeats, cytodiagnosis, p53 gene, head and neck carcinoma

## Abstract

Oesopharyngeal brush (OPB) sampling with cytological analysis can yield exfoliated cells from asymptomatic tumours of the upper aero-digestive tract and the oesophagus. In this study, we compared cytological evaluation and molecular analysis for the detection of exfoliated cancer cells sampled with an OPB. A total of 56 patients with a known unique head and neck squamous cell carcinoma (HNSCC) and five healthy controls were enrolled prospectively. Exfoliated cells from these 61 patients were collected with an OPB before initial endoscopy. p53 mutations and UT 5085 microsatellite instability (MI) were analysed in the HNSCC tumour, lymphocytes and the corresponding OPB DNA samples. p53 mutations and UT5085 MI were detected in 31 out of 56 and 14 out of 56 HNSCC, respectively, but not in any of the five controls. Direct sequencing of p53 was able to detect mutations in OPB DNA in only two out of 29 patients harbouring a p53-mutated primary tumour. Microsatellite instability was detected in OPB DNA of 11 out of 13 informative (bandshift detected in tumour) patients, whereas cytological analysis detected abnormal cells in only six of the same 13 patients (*P*=0.03). In informative patients, all positive OPB samples at cytological analysis were also positive at molecular analysis of UT5085, and both analyses confirmed the two negative samples. Molecular analysis of OPB from eight uninformative patients and from five healthy controls were all negative. OPB sampling with MI-based molecular analysis could be efficient for early detection of recurrent HNSCC. This result prompts us to use other microsatellite markers in order to maximise the percentage of informative patients.

With 23 000 new cases annually, the incidence of head and neck squamous cell carcinoma (HNSCC) in France is one of the highest in the world (Hill *et al*, 1997) because of smoking and alcohol consumption (IARC, 1986,^1988^). Early detection of HNSCC could improve therapeutic results and reduce morbidity and mortality. Loco-regional relapses occur in more than 30% of HNSCC patients and mostly during the first 2 years after treatment (McCarty and Million, 1994; Haas *et al*, 2001). Salvage treatments can only be effective if recurrences are detected rapidly. The incidence of second primary metachronous HNSCC attains 2–4% annually and these lesions also need to be detected early (Panosetti *et al*, 1989; Erkal *et al*, 2001). However, after surgery and/or radiotherapy, clinical and radiological surveillance is difficult because of anatomical modifications and fibrosis. Both clinical and nasofibroscopic examinations are only limited to the upper part of the aero-digestive tract (Boysen *et al*, 1992). Cytological screening (Ogden, 1997) has been developed by collecting saliva with or without an oral mucosal brush. Recently, an encapsulated oesopharyngeal brush (OPB) technique for cytological analysis was developed to detect recurrent and metachronous oesophageal carcinomas (Leoni-Parvex *et al*, 2000). Oesopharyngeal brush has also been shown to be capable of collecting exfoliated cells from oral or pharyngeal carcinoma. This technique, whose sensitivity and specificity are high, appears to be safe and minimally invasive.

Head and neck squamous cell carcinoma is a multistep genetic process (Califano *et al*, 1996; Mao *et al*, 1996a) during which proto-oncogenes are activated and tumour-suppressor genes are inactivated. Tri- and tetranucleotide microsatellite instability (MI) is characterised by the insertion or deletion of one or more repeat units of three or four nucleotides. These sporadic microsatellite mutations are independent of mutations in mismatch repair genes and reflect DNA damage caused by exposure to carcinogens in tobacco smoke (Slebos *et al*, 2002). Microsatellite instability is regarded as a clonal marker of HNSCC (Mao *et al*, 1994; Coulet *et al*, 2000), which can be used to detect small amounts of tumour DNA (Forastiere *et al*, 2001). Microsatellite instability is a qualitative variable that can be detected by PCR-based techniques such as bandshift assays. The UT5085 marker currently appears to be the most informative marker of MI in HNSCC (Coulet *et al*, 2000). The p53 tumour-suppressor gene is mutated in more than 50% of HNSCC (Temam *et al*, 2000). Different PCR techniques have been described for the detection of p53 mutations. The sensitivity and the reproducibility of PCR have recently been reviewed (van Houten *et al*, 2000). The most sensitive techniques, including cDNA cloning and specific hybridisation, are very time consuming and are difficult to apply in routine practice.

Molecular approaches have rarely been compared with conventional cytological analysis in HNSCC (Mao *et al*, 1994). The aim of the present study was to test the OPB for the detection of tumour cells from the head and neck region, and to compare conventional cytological evaluation with molecular analysis using two techniques: analysis of MI at the UT5085 locus and direct sequencing of p53.

## MATERIALS AND METHODS

### Patients and sample collection

Patients were referred to the Institut Gustave Roussy between July 1999 and June 2000. All patients underwent OPB sampling before panendoscopy with oesophagoscopy, which excluded a second oesophageal tumour. Oesopharyngeal brush sampling was performed, as previously described (Leoni-Parvex *et al*, 2000). An abrasive sponge wrapped in a capsule (Oesotest®, Biodessa, France) was first swallowed and then withdrawn with a string. It was shaken in a saline fluid that was centrifuged over 10 min at 3000 **g**. The supernatant was discarded and the cell pellet was retained. Part of this pellet was smeared onto three glass slides and fixed in alcohol. Slides were stained using the Papanicolaou method for cytological evaluation and the rest of the sediment was frozen for biological studies. The institutional review board approved the study, and informed consent was obtained from all subjects.

In total, 56 fresh-frozen primary HNSCC biopsy samples were collected during panendoscopy from 56 patients. All samples were diagnosed as invasive HNSCC and samples containing more than 70% of tumour cells were selected in order to avoid microdissection. Normal mucosa biopsy specimens were also collected during panendoscopy from five healthy patients, who were heavy smokers and drinkers, but free of HNSCC. At the time of the initial diagnosis, fresh blood was collected from patients in EDTA tubes and lymphocytes were separated for use as normal DNA.

### Cytological criteria

Cytological specimens were considered positive if there were low- or high-grade squamous dysplastic cells or squamous carcinoma cells, at least on one slide. This definition of positivity was based on oeosophageal cytological criteria proposed by Shu (1983) and used by others (Leoni-Parvex *et al*, 2000). The cytological evaluation was considered negative only when there was no significant cytonuclear atypia in squamous cells and if the majority of cells were of the intermediate type.

### Analysis of UT5085 microsatellite in tumour DNA and OPB samples

DNA was extracted with the QIAamp Blood Kit (Qiagen, Courtaboeuf, France). DNA quality was checked with GeneQuant II (Amersham Pharmacia Biotech, Cambridge, UK). The UT5085 tetranucleotidic microsatellite was amplified with primers referenced in the GenBank sequence database (accession number GDB 309286), as described (Coulet *et al*, 2000): (a) UT5085A, 5′-AAAGTGGGGATAAGGCAGC-3′; and (b) UT5085B, 5′-AGATGCACAACACATACACG-3′. The primer (a) UT5085A was labelled in blue by 6-carboxy-fluorescein (FAM). Amplification was performed in a 20-*μ*l reaction volume with 0.5 U HotStart *Taq* DNA Polymerase (Qiagen, Courtaboeuf, France), 0.2 mM deoxynucleotide triphosphate, 3.2 pmol of each forward and reverse primer, 2 mM MgCl2, and 2 *μ*l of 10 × Qiagen buffer. A total of 100 ng of DNA was used as a template for each sample. The mixtures were denatured for 20 min at 95°C followed by 35 cycles at 95°C for 30 s, 54°C for 30 s and 72°C for 30 s. A final elongation was performed for 10 min at 72°C. Amplified products were run in polyacrylamide sequencing gels, ELLIOSEQ GEL (Laboratoires Ellios Bio Media, Paris, France) mixed with 0.5 *μ*l of GENESCAN-500 ROX (Applied Biosystems, Inc., Warrington, UK) and 2.5 *μ*l of formamide blue after a 2 min denaturing step at 94°C. Polymerase chain reaction (PCR) products were detected on the gel by laser fluorescence on the ABI prism 377 DNA sequencer (Applied Biosystems, Inc.). Data were analysed by Genescan analysis (Applied Biosystems, Inc.).

All tumours and corresponding lymphocytes were amplified to select unstable UT5085 samples. A normal mucosa biopsy specimen was used for the five control patients. Microsatellite instability (MI) was defined as the presence of a new pattern in at least one extra band in tumour DNA compared to lymphocyte DNA. Patients with UT5085 MI in tumours were defined as informative patients.

Oesopharyngeal brush material corresponding to all shifted tumours was then amplified and coanalysed with tumour and lymphocyte DNA to compare the different microsatellite patterns. We also studied OPB samples from eight uninformative tumours and from the five control subjects. Two observers assessed all samples independently and borderline cases were repeated and evaluated by densitometry.

### p53 mutation detection in tumour DNA and OPB samples

Exons 2–11 were screened for mutations by denaturing high-performance liquid chromatography (DHPLC) using the Wave® (Transgenomic, Inc., Omaha, USA). Tumours with a variant chromatographic pattern were specifically amplified by PCR and both strands were sequenced for each variant exon using the ABI prism 377 DNA sequencer (Applied Biosystems, Inc.) with the Big Dye Terminator sequencing kit (Applied Biosystems, Inc). Data were analysed by Sequencing Analysis Software (Applied Biosystems, Inc). All mutations were confirmed twice on both strands.

Oesopharyngeal brush material from patients harbouring a p53-mutated tumour was analysed to search for the same p53 mutation. We used direct sequencing of the putative mutated exon under the same conditions as in the tumour.

### Statistical analysis

*χ*^2^ tests were used to compare qualitative variables.

## RESULTS

### Population

In total, 56 patients with a unique HNSCC were prospectively entered into the study. Primary tumour sites were: the oral cavity, oropharynx, larynx and hypopharynx in 13, 23, 12 and eight cases, respectively. There were 18 T1-2 and 38 T3-4 lesions (Table 1

**Table 1:** Clinical and biological characteristics of the 56 HNSCCs, according to cytological and biological study of pharyngo-oesophageal brushing (Oesotest®)

			**Cytology**	**Microsatellite UT5085**		**p53 mutations**	
**Pts no.**	**Tumour site**	**T stage**	**Oesotest**	**Tumour**	**Oesotest**	**Tumour**	**Oesotest**
1	Oropharynx	T4	Carcinoma	Shift	Shift	Mut	Neg
2	Oropharynx	T3	Neg	Shift	Shift	Mut	Mut
3	Oropharynx	T4	Neg	Shift	Shift	Mut	Neg
4	Larynx	T2	Carcinoma	Shift	Shift	Mut	Neg
5	Larynx	T4	Carcinoma	Shift	Shift	Mut	Neg
6	Hypopharynx	T2	Neg	Shift	Shift	Mut	Neg
7	Oral cavity	T4	High-grade dysplasia	Shift	Shift	Mut	Neg
8	Oropharynx	T4	Carcinoma	Shift	Shift	Wild	ND
9	Oropharynx	T2	Carcinoma	Shift	Shift	Wild	ND
10	Hypopharynx	T4	Neg	Shift	Shift	Wild	ND
11	Oral cavity	T4	Neg	Shift	Shift	Wild	ND
12	Larynx	T3	Neg	Shift	Neg	Mut	Neg
13	Oropharynx	T4	Neg	Shift	Neg	Wild	ND
14	Oropharynx	T3	Neg	Shift	No DNA	Mut	No DNA
15	Hypopharynx	T4	Carcinoma	Neg	Neg	Mut	Mut
16	Oropharynx	T3	Carcinoma	Neg	Neg	Mut	Neg
17	Oropharynx	T4	Neg	Neg	ND	Mut	Neg
18	Oropharynx	T3	Neg	Neg	ND	Mut	Neg
19	Oropharynx	T4	High-grade dysplasia	Neg	ND	Mut	Neg
20	Oropharynx	T4	Carcinoma	Neg	ND	Mut	Neg
21	Oropharynx	T2	Neg	Neg	ND	Mut	Neg
22	Oropharynx	T2	Neg	Neg	ND	Mut	Neg
23	Oropharynx	T3	Low-grade dysplasia	Neg	ND	Mut	Neg
24	Oropharynx	T2	Carcinoma	Neg	ND	Mut	Neg
25	Larynx	T3	Carcinoma	Neg	Neg	Mut	Neg
26	Larynx	T3	Neg	Neg	Neg	Mut	Neg
27	Larynx	T4	Neg	Neg	ND	Mut	Neg
28	Larynx	T2	Low-grade dysplasia	Neg	ND	Mut	Neg
29	Hypopharynx	T2	Carcinoma	Neg	ND	Mut	Neg
30	Hypopharynx	T4	Neg	Neg	Neg	Mut	Neg
31	Hypopharynx	T1	Neg	Neg	Neg	Mut	ND
32	Hypopharynx	T4	Neg	Neg	ND	Mut	Neg
33	Oral cavity	T4	Neg	Neg	ND	Mut	Neg
34	Oral cavity	T4	Neg	Neg	Neg	Mut	Neg
35	Oral cavity	T3	Neg	Neg	Neg	Mut	Neg
36	Oral Cavity	T3	Neg	Neg	ND	Mut	Neg
37	Oropharynx	T1	Carcinoma	Neg	Neg	Wild	ND
38	Oropharynx	T3	Neg	Neg	ND	Wild	ND
39	Oropharynx	T2	Neg	Neg	ND	Wild	ND
40	Oropharynx	T2	Neg	Neg	ND	Wild	ND
41	Oropharynx	T3	Neg	Neg	ND	Wild	ND
42	Oropharynx	T2	Neg	Neg	ND	Wild	ND
43	Oropharynx	T1	Neg	Neg	ND	Wild	ND
44	Larynx	T4	Neg	Neg	ND	Wild	ND
45	Larynx	T2	Low-grade dysplasia	Neg	ND	Wild	ND
46	Larynx	T3	High-grade dysplasia	Neg	ND	Wild	ND
47	Larynx	T4	Neg	Neg	ND	Wild	ND
48	Larynx	T1	Neg	Neg	Neg	Wild	ND
49	Hypopharynx	T4	Neg	Neg	ND	Wild	ND
50	Oral cavity	T2	Neg	Neg	ND	Wild	ND
51	Oral cavity	T4	Neg	Neg	ND	Wild	ND
52	Oral cavity	T4	Neg	Neg	ND	Wild	ND
53	Oral cavity	T4	High-grade dysplasia	Neg	ND	Wild	ND
54	Oral cavity	T4	Carcinoma	Neg	ND	Wild	ND
55	Oral cavity	T3	Neg	Neg	ND	Wild	ND
56	Oral cavity	T2	Neg	Neg	ND	Wild	ND

Neg=negative; ND=not done; shift=microsatellite instability at UT5085; Mut=p53 mutation; Wild=p53 wild type.

). Five healthy control patients, who were heavy smokers and drinkers but free of HNSCC, were also studied.

### Molecular markers in primary tumours

UT5085 tetranucleotide MI was analysed in all tumours and corresponding lymphocytes (Table 1). Tumours were informative (i.e. exhibited a bandshift) in only 14 out of 56 (25%) patients and not in five controls. p53 mutations were detected in 31 out of 56 (55%) tumours. Of the p53 mutations, 21 (68%) were missense mutations, nine (29%) were nonsense mutations and one (3%) was a silent mutation. Details of p53 mutations are shown in Table 2

**Table 2:** p53 mutations in 31 patients with HNSCC

Patients no.	Exon	Codon^a^	Sequence	Amino acid
28	4	35	Deletion 1 bp	Frameshift
35	4	111	CTG→CAG	L→Q
18	5	125	ACG→AGG	T→R
27	5	126	TAC→TGC	Y→C
31	5	134	TTT→GTT	F→V
30	5	146	TGG→TAG	W→Stop
36	5	179	CAT→CTT	H→L
34	5	183	TCA→TGA	S→Stop
3	5	187	GGT→GTT	G→V
1	6	193	CAT→CTT	H→L
12	6	204	GAG→TAG	E→Stop
7	7	245	GGC→GAC	G→V
2	7	247	AAC→AAT	N→N
24	7	248	CGC→CTG	R→L
19	7	248	CGG→CAG	R→L
15	7	257	Insertion 1 bp	Frameshift
26	8	270	TTT→TCT	F→S
32, 33, 20	8	273	CGT→TGT	R→C
14	8	273	CGT→CAT	R→H
23	8	275	TGT→TTT	C→F
4, 29, 17, 21	8	282	CGG→TGG	R→W
5	9	319	AAG→TAG	K→Stop
16	9	321	Deletion 1 bp	Frameshift
25	9	331	CAG→TAG	Q→Stop
6	9	327 or 328	Insertion 1 bp	Frameshift
22	10	Intron 11	17708 A→T	Silent

a First codon of the frameshift mutation. bp=base pair.

. There was no relation between molecular analysis results and the T stage or tumour location. No significant correlation was found between p53 mutations and UT5085 MI: nine out of 14 (64%) tumours with UT 5085 MI were p53 mutated *vs* 22 out of 42 without MI (52%; *P*=0.6).

### Cytological analysis and molecular markers in OPB samples

All cytological samples were available for interpretation. Of the 56 OPB DNA samples, 20 (36%) were positive at cytological examination: 13 with squamous carcinoma cells and seven with dysplastic cells (Table 1). There was no relation between the results of the cytological analysis and the T stage or tumour location. Oesopharyngeal brush cytology was normal in all five control subjects.

**Figure 1:**
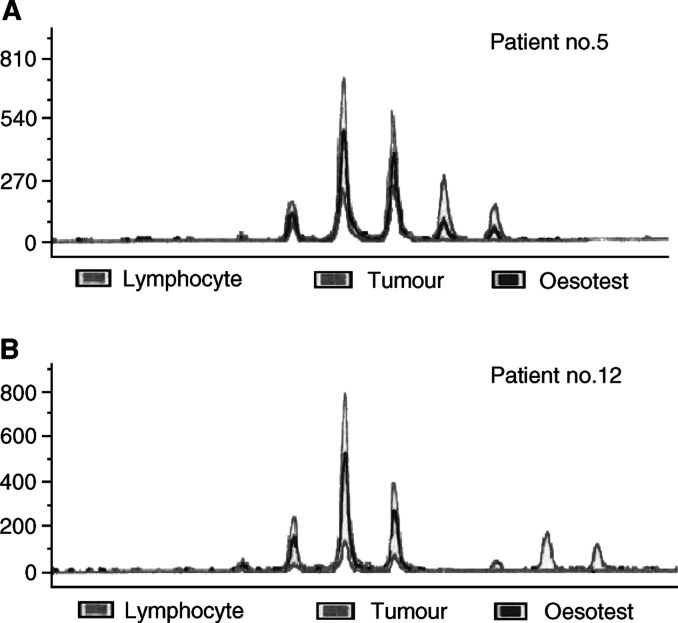
Microsatellite UT5085 of paired tumour, lymphocyte and pharyngo-oesophageal brushing (Oesotest®). (**A**) Patient number 5 who had an informative tumour (bandshift) and a positive brushing with the same pattern (same bandshift as tumour). (**B**) Patient number 12 who had an informative tumour and a negative brushing.

Oesopharyngeal brush DNA was available for the evaluation of UT5085 tetranucleotide MI in 13 out of 14 informative patients. In these informative patients, a bandshift, similar to that observed in the primary tumour, was detected in 11 out of 13 (85%) OPB DNA samples (Figure 1A

). In the same samples, tumour cells were detected at cytological analysis in only six out of 13 (46%) patients. In informative patients all positive OPB DNA samples at cytological evaluation were also positive at molecular analysis of UT5085 (Table 3

**Table 3:** Comparison of cytological examination with Papanicolaou staining and microsatellite analysis of pharyngo-oesophageal brush samples for the 13 patients whose tumour exhibited an MI at UT5085

	Cytology		
	Positive^a^	Negative	Total
*Microsatellite UT5085*			
Shift	6	5	11
No shift	0	2	2
Total	6	7	13

Molecular analysis was more sensitive than cytological study (*P*=0.03).

a Positive=low- or high-grade squamous dysplasia cells or squamous cells carcinoma.

), and the two samples found to be negative at molecular analysis were also negative at cytological analysis (Figure 1B). In this subgroup, the molecular analysis with the UT5085 marker was more sensitive than the cytological analysis (*P*=0.03). Molecular analysis with UT5085 was negative for the 13 controls: five healthy subjects and eight uninformative patients.

In the subgroup of 31 patients exhibiting a p53 mutation, DNA was available for 29 OPB samples. Direct sequencing of p53 was able to detect tumour DNA in OPB samples from only two out of 29 (7%) samples, whereas cytological evaluation was positive in 13 out of 29 OPB samples (45%; Table 4

**Table 4:** Comparison of cytological examination with Papanicolaou staining and p53 direct sequencing of pharyngo-oesophageal brushing for the 29 patients with p53 mutated tumours

	Cytology		
	Positive^a^	Negative	Total
*Mutated p53*			
Yes	1	1	2
No	12	15	27
Total	13	16	29

a Positive=low- or high-grade squamous dysplasia cells or squamous cells carcinoma.

). Of these 13 OPB DNA samples, 12 were positive at cytological analysis and negative at direct sequencing of p53.

## DISCUSSION

Oesopharyngeal brush with cytological analysis was mainly developed in China for mass screening of oesophageal carcinoma (Dowlatshahi *et al*, 1978; Jaskiewicz *et al*, 1987). The sensitivity of this technique ranges from 40 to 70% and its specificity from 90 to 99%. Several teams have used the OPB to detect second primary tumours during the follow-up of patients treated for HNSCC, because clinical examination is difficult and is limited to the upper part of the aero-digestive tract. The OPB has indeed proved very sensitive for the detection of new primary lesions. In the study by Pellanda *et al* (1999), none of the 254 patients with negative OPB cytology developed an oesophageal tumour. Among the 25 asymptomatic patients with positive OPB cytology, a premalignant or early oesophageal tumour was detected in 20 cases. Interestingly, in the same study 10 clinically unsuspected oral or pharyngeal carcinoma were also detected. These data suggest that exfoliated cells from the upper part of the aero-digestive tract can be swallowed and sampled with the OPB. This technique for collecting tumour cells could therefore be as efficient as saliva sampling with the added advantage of allowing access to the hypopharynx and oesophagus. In the present study, the OPB was used before endoscopy and the initial biopsy. The oesophagoscopy excluded a second primary tumour of the oesophagus.

The aims of our study were two-fold: (i) to test the capacity of the OPB in detecting tumour cells from inaccessible parts of the head and neck region and (ii) to compare cytological *vs* molecular analysis for the detection of tumour exfoliated cells in a population of 56 HNSCC patients.

We confirm that tumour cells exfoliated from the head and neck region can be sampled in the oesophagus. It is noteworthy that OPB detected tumour cells and/or tumour DNA in seven out of 12 laryngeal carcinoma in the oesophagus, as shown in Table 1. Perhaps, this easy and low-invasive technique could be tested in bronchial carcinoma.

The results of the molecular analysis of OPB samples are directly linked to the technique used. In this study, we tested two different techniques: MI analysis and p53 direct sequencing. Microsatellite alterations on selected tetranucleotide markers are common in non-small-cell lung, head and neck, and bladder carcinoma (Mao *et al*, 1994). The generation of a novel allele through the insertion or deletion of a short tandem repeat creates a new band that is only present in neoplastic cells. This new band is easier to detect than a subtle change as in loss of heterozygosity. Our results show that for informative patients, molecular analysis with UT 5085 appears to be more sensitive than cytology for the detection of exfoliated tumour cells: abnormal DNA was detected in 11 out of 13 OPB samples, compared to six out of 13 abnormal or tumour cells detected at cytology (*P*=0.03). These results will prompt us to use panels of microsatellite markers, in order to increase the sensitivity of the genetic analysis. Using a panel of 23 informative microsatellite markers, Spafford *et al* (2001) found a much higher rate (57 *vs* 25%) of MI in HNSCC patients.

Mao *et al* (1994), using a panel of nine tri- and tetranucleotide markers, demonstrated the interest of MI as clonal markers for the detection of HNSCC cells. They detected identical microsatellite alterations in corresponding serum, sputum and surgical margins. In patients treated for bladder carcinoma, microsatellite analysis of urine sediment was found to be more efficient than cytological analysis: primary bladder cancer was identified in 19 out of 20 patients at microsatellite analysis, whereas only nine out of 18 lesions were detected at cytological analysis (Mao *et al*, 1996b). Thus, in these informative patients,the difference between the two techniques (conventional cytological analysis and microsatellite analysis) is similar to that found in the present study. Studying pretreatment oral rinses and swab samples from HNSCC patients, Spafford *et al* detected MI in 24 out of 25 (96%) informative patients. Like most authors, they did not, however, compare microsatellite detection and cytology (Spafford *et al*, 2001). Our study shows that microsatellite-based molecular analysis can be positive in cytologically negative samples.

In our experience, the bandshift profile detected in OPB samples must be compared with that of the primary tumour. DNA extracted from exfoliated cells can undergo degradation and it is mandatory that bandshifts be distinguished from artefacts. Microsatellite-based molecular analysis should be efficient for early detection of recurrences of a tumour that has already been analysed. However, unlike cytology, this technique will be difficult to use for the detection of second primary tumours, which do not necessarily share the same genetic alterations.

Conversely and based on the concept of field cancerisation, we cannot exclude the possibility that cells other than those of the primary tumour share the same genetic alterations. Mucosa exhibiting a normal appearance in patients with early cancer or premalignant lesions has been shown to harbour occult microsatellite alterations (Califano *et al*, 1996). However, we have evidenced that the molecular analysis of UT5085 in OPB samples from eight patients with an uninformative tumour and from five heavy smokers free of HNSCC were all negative.

Although we found a 55% rate of p53 mutations in primary tumours, which is similar to that usually observed in HNSCC, only a 7% rate of p53 mutations was found in OPB samples from patients with p53 mutations. As shown by Boyle *et al* (1994), p53-mutated cells are present in the saliva of HNSCC patients. They used another technique, namely cloning of p53 sequences followed by hybridisation with specific radio-labelled oligonucleotide probes (van Houten *et al*, 2000). Two independent papers have demonstrated the clinical relevance of that technique by investigating surgical margins of tumours from head and neck cancer patients (Brennan *et al*, 1995; Partridge *et al*, 2000). However, this sensitive and clinically validated technique is very time consuming and cannot be used in routine practice. Our results also confirm that direct sequencing of p53 is not a sensitive technique for detecting small amounts of tumour DNA. In the present work, we found no association between UT 5085 MI and p53 mutations in the primary tumour. This is in contrast with the results reported by Ahrendt *et al* (2000) in non-small-cell lung cancer.

The MI rate for one marker is low. As this study clearly indicates that microsatellite-based techniques could be efficient for detecting early recurrences, we could increase the number of MI markers, as already performed by Spafford *et al* (2001), in order to maximise the rate of informative patients.

Other molecular markers have been tested for the diagnosis of minimal residual disease in HNSCC. A panel of molecular markers based on promoter hypermethylation of selected genes (Sanchez-Cespedes *et al*, 2000; Rosas *et al*, 2001) could be of particular interest as well as mutations in mitochondrial DNA (Fliss *et al*, 2000; Sanchez-Cespedes *et al*, 2001; Ha *et al*, 2002). This study on patients with known HNSCC is the first step towards a molecular diagnosis test for the follow-up of patients with HNSCC. In the near future, these cytodiagnosis techniques associated with molecular analysis could be tested in populations of heavy drinkers and smokers. Patients with asymptomatic tumours detected with these molecular markers could thus benefit from early treatment.
